# Overestimation of corneal endothelial cell density by automated method in glaucomatous eyes with impaired corneal endothelial cells

**DOI:** 10.1007/s10792-021-02008-4

**Published:** 2021-09-05

**Authors:** Mayumi Minami, Etsuo Chihara

**Affiliations:** 1 Sensho-kai Eye institute, Minamiyama 50-1, Iseda, Uji, Kyoto, 611-0043 Japan; 2 Minami Eye Clinic, Yokaichi Midorimachi 1-7, Higashi-omi, Shiga, 527-0023 Japan

**Keywords:** Corneal endothelial cell density, Non-contact specular microscopy, Center method, Automated method, Glaucoma

## Abstract

**Purpose:**

To determine between-method differences in corneal endothelial cell parameters using center and automated methods of non-contact specular microscopy (CellCheck software of Konan, Inc.) in glaucomatous eyes.

**Methods:**

We analyzed the central corneal endothelial cell density (ECD) of 245 glaucomatous eyes using center (ECD-Ce) and automated methods (ECD-Au). Based on the ECD-Ce, we allocated subjects to Groups 1 to 10 (at 250 cells/mm^2^ intervals) and evaluated the ECD, coefficient of variation in cell area (CV), and percentage of hexagonal cells (HEX).

**Results:**

There was a close correlation (*r* = 0.91) between the ECD values measured using both methods. However, ECD-Au were significantly higher than those measured by the center method when ECD-Ce was less than 2500 (in Groups 1 to 8; *P* < 0.001 to *P* = 0.006). The regression equation of (ECD-Au—ECD-Ce) = 1028–0.397*ECD-Ce shows greater deviation in eyes with lower ECD, and this difference became 0 when ECD -Ce was 2593 cells/mm^2^. None of the 44 subjects with an ECD-Ce of < 1000 cells/mm^2^ recorded an ECD-Au < 1000 cells/mm^2^. Compared with the center method, the automated method had higher and lower median CV and HEX values, respectively (*P* < 0.001). The between-method differences in both CV and HEX were negatively correlated with ECD-Ce (*r* = −0.49, *P* < 0.001 and *r* = −0.25, *P* < 0.001, respectively).

**Conclusion:**

The automated method of the CellCheck software overestimates ECD in eyes with lower ECD values and may overlook risk of corneal decompensation.

## Introduction

Corneal endothelial cells have ion transport systems that counteract water imbibition by the corneal stroma, and therefore maintain corneal clarity. In humans, corneal endothelial cells do not proliferate. Severe endothelial cell loss can cause bullous keratopathy. Currently, there are multiple glaucoma treatment methods; among them, trabeculectomy and tube shunt surgery may affect the corneal endothelium [1]. Therefore, it is important to accurately assess corneal endothelial cell parameters to evaluate the risk of corneal decompensation and choose appropriate glaucoma treatment methods. Endothelial cell density (ECD) is an important parameter for assessing endothelial function and health. Polymegathism, determined by the coefficient of variation in cell area (CV), and pleomorphism, represented by the percentage of hexagonal cells (HEX), are other important parameters that indicate stress to the endothelium [2].

The Food and Drug Administration of USA recommends the center method of specular microscopy as the “gold standard,” and it is used by virtually every professional reading center [3, 4]. On the other hand, the automated method is widely used in daily clinical practice because of its simplicity. When we use ECD data utilizing the automated method (ECD-Au), we must be careful that the ECD-Au may overestimate the ECD in glaucomatous eyes [5], post-Descemet stripping endothelial keratoplasty eyes [6], and eyes with low corneal ECD [5, 7]. As far as glaucoma eyes are concerned, there is only one report on overestimation of ECD and concerns for overestimation of ECD are yet to be well recognized.

Assuming ECD values obtained using the center method (ECD-Ce) approximate the real data, it is possible to evaluate the reliability of the automated method. In this study, we compared endothelial parameters of ECD, CV, and HEX in glaucomatous eyes obtained using both methods. To the best of our knowledge, there has been no study on the specific ECD range where a significant between-method difference becomes apparent. Therefore, to determine the critical ECD level, we provided a scatterplot to study the correlation between the difference between ECD-Ce and ECD-Au, and we allocated subjects into groups based on the ECD-Ce (250 cells/mm^2^ intervals) and analyzed between-method differences.

## Materials and methods

This was a retrospective comparative study. The primary outcome measures were the ECD, HEX, and CV. We enrolled patients with a clear central cornea, allowing the acquisition of high-quality images of the corneal endothelium. We analyzed the eyes with a minimum of 30 contiguous countable cells.

We excluded images of poor quality due to halation, defocus, and darkness. Moreover, we excluded subjects with active anterior uveitis with cells or flare in the anterior chamber, active infectious conjunctivitis, keratoplasty history, severe corneal erosion, epithelial defects, and parenchymal opacity. We included patients with eyes with pterygium and corneal neovascularization if there was no encroachment into the image area.

We conducted a retrospective medical record review of central corneal endothelial images obtained using a non-contact specular microscope (Noncon Robo FA-3809II ver.4.05; Konan Medical Inc. Hyogo, Japan), which did not have an option to select patterns of cell size. We evaluated one image from each of the 260 glaucomatous eyes of 191 consecutive outpatients who visited the glaucoma service of Sensho-kai between September 2019 and January 2020; photographs of the endothelium were obtained and stored in a memory of device independently by 11 technicians blinded to the study between September 2010 and December 2019. As a control, we studied 36 eyes of 36 cataract patients who visited our institute in February 2020. These patients did not have a history of intraocular surgery, corneal disease, or intraocular inflammation.

We outputted corneal endothelial images stored in the computer memory, and an ophthalmologist (MM) reviewed each image and analyzed the data using both methods. For images obtained using the center method, the maximum number of contiguous cells was counted within the largest viewable field by dotting each cell’s center.

We classified the subjects into small groups based on ECD-Ce (group intervals of 250 cells/mm^2^). Moreover, we analyzed the CV, HEX, number of analyzed cells (NUM), and central corneal thickness (CCT) obtained using specular microscopy. CV values were presented as (standard deviation of cell areas, μm^2^/mean cell area, μm^2^) × 100. Moreover, we recorded the following for each participant: age, gender, Goldman applanation tonometric intraocular pressure (IOP) on the measurement, and glaucoma classification.

This study was approved by the Institutional Review Board of the Sensho-kai Eye Institute and followed the tenets of the Declaration of Helsinki. Written informed consent was obtained from all the participants.

## Statistical analysis

All statistical analyses were performed using Bell Curve Excel Tokei 2016 (SSRI, Tokyo, Japan), JMP (SAS Institute Inc., Cary, NC, USA), and StatView5.0 (SAS Institute, Cary, NC) software. We assessed between-method agreements in the ECD, CV, and HEX values using the Mann–Whitney U test. Moreover, we used the Bland–Altman method to assess the between-method agreements of the ECD values. The Spearman test was used to evaluate the correlation coefficients between the evaluated data. Statistical significance was set at *P* < 0.05.

## Results

**Fig. 1:**
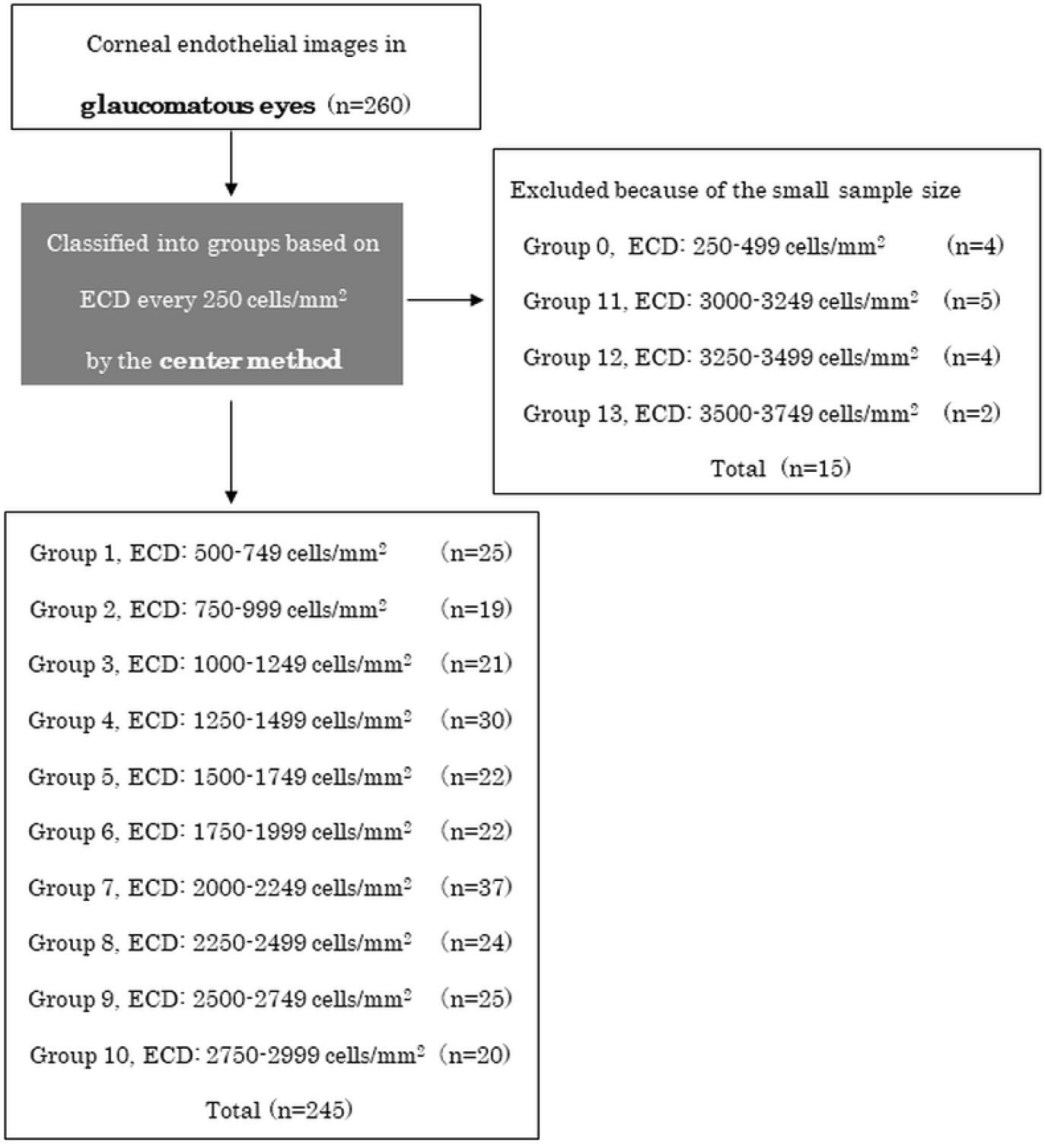
Flow diagram of the study design

Figure 1 shows the flow diagram of the study design. Two hundred and sixty eyes of 191 patients were classified into 14 groups (Groups 0–13) based on ECD-Ce at 250 cells/mm^2^ intervals. We excluded 15 subjects from four groups due to the small sample sizes of the subgroups. Consequently, we included 245 eyes from 183 patients in the final analysis.

Intra-individual reproducibility of data was studied in six right eyes of six control subjects by repeating examinations five times, and the intraclass correlation coefficient (ICC) and 95% confidence interval (CI), and the coefficient of variation (%) were calculated and is listed in Table 1. The ICC (1,5) of the ECD, CV, and HEX by the automated method (0.980, 0.777, and 0.706, respectively) and ECD-Ce (0.973) were greater than 0.7, showing good reproducibility, while those for HEX (ICC (1,5) = 0.514) and CV (ICC (1,5) = 0.412) by the center method showed fair reproducibility (Table 1).

**Table 1:** Reproducibility of data assessed by intraclass correlation values and coefficient of variation

	ICC (1,5)	95% CI	CV (%)
Center method			
ECD	0.973	0.910–0.996	5.18
CV	0.412	−0.987–0.908	12.71
HEX	0.514	−0.644–0.924	14.91
Automated method			
ECD	0.980	0.936–0.997	3.16
CV	0.777	0.297–0.965	8.76
HEX	0.706	0.072–0.953	9.25

*ICC* intraclass correlation coefficients, *95% CI* 95% confidence interval, *ECD* endothelial cell density, *CV* coefficient of variation in cell area, *HEX* percentage of hexagonal cells

Table 2 presents the demographic characteristics of the glaucoma and control groups. The type of glaucoma based on guidelines [8] was 78 primary open-angle glaucoma, 16 primary angle-closure glaucoma, 49 neovascular glaucoma, 42 exfoliation glaucoma, and 8 childhood glaucoma. Two eyes with traumatic glaucoma underwent blunt trauma 12 months and 7 years before the measurement date, respectively.

**Table 2:** Demographic data of glaucoma and control subjects

Number	245 eyes of 183 patients	36 eyes of 36 control subjects
Eye, *n* (%)		
Right	118 (41)	36 (100)
Left	127 (59)	0
Age (*y*), median (IQR)	70 (58–76)	71 (67–76)
Sex, n (%)		
Male	143 (58)	18 (50)
Female	102 (42)	18 (50)
IOP (mmHg), mean ± SD	19.5 ± 10.2	17.8 ± 2.4
Diagnosis,	N	
1. Primary glaucoma	84	All 36 have cataract
POAG (broad definition)*	78	
PACG	16	
2. Secondary glaucoma	153	
Neovascular glaucoma	49	
Exfoliation glaucoma	42	
Malignant glaucoma	3	
Posner Schlossman syndrome	2	
Harada disease	2	
Steroid glaucoma	2	
Iridocorneal endothelial syndrome	2	
Glaucoma secondary to vitreous surgery	2	
Traumatic glaucoma	2	
Microphthalmia	2	
Other uveitis, undetermined or mixed	35	
3. Childhood glaucoma	8	

* POAG includes both ocular hypertension and normal tension glaucoma

*IQR* interquartile range, *IOP* intraocular pressure, *SD* standard deviation, *POAG* primary open-angle glaucoma, *PACG* primary angle-closure glaucoma

**Fig. 2:**
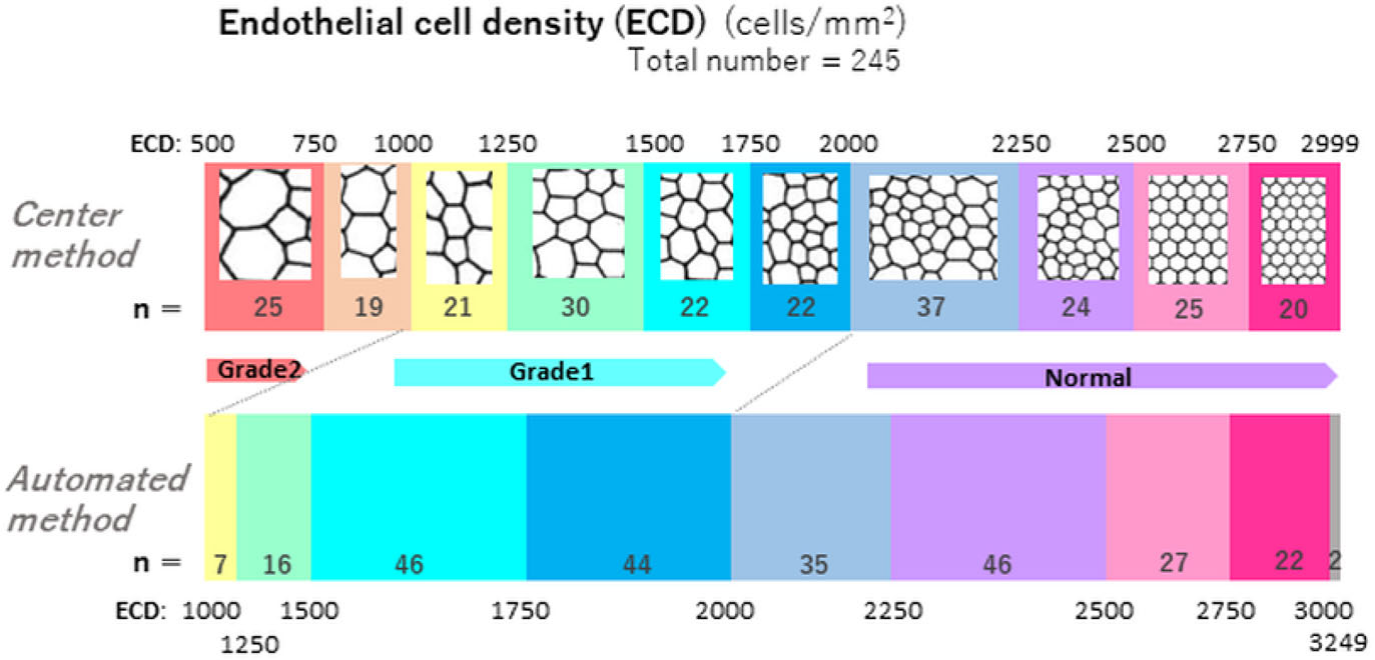
Breakdown of each group graded by corneal endothelial cell density (ECD) measured using the center (superior column) and automated method (inferior column). The automated method overestimated the ECD value of the damaged eye. Forty-four subjects which were classified into Grade 2 (ECD-Ce: 500–999 cells/mm^2^) did not record an ECD < 1000 cells/mm^2^ using the automated method

**Fig. 3:**
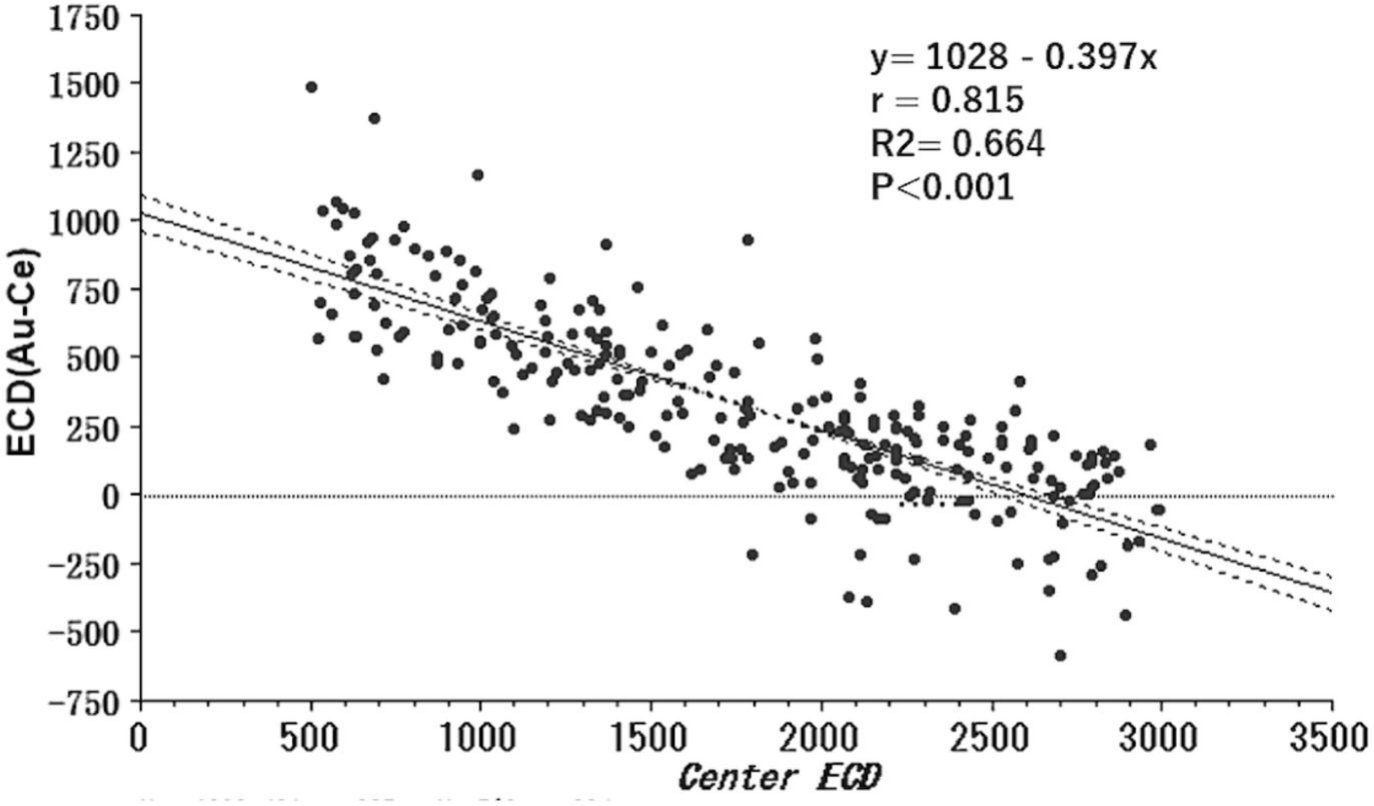
Scatter plot showing difference between (ECD-Au – ECD-Ce) and ECD-Ce with regression line and 95% confidence interval. The difference becomes zero when the ECD-Ce = 2593 cells/mm^2^

Figure 2 shows the number of participants in each group. Corneal endothelial damage was graded according to the Japanese corneal society guidelines [9] as follows: normal, ≥ 2000 cells/mm^2^; grade 1, 1000–2000 cells/mm^2^; and grade 2, 500–1000 cells/mm^2^. When the ECD was low, ECD-Au was greater than ECD-Ce. None of the 245 eyes was grade 2 using the automated method (ECD-Au less than 1000 cells/mm^2^), while 44 eyes were classified as Grade 2 (ECD-Ce 500–1000 cells/mm^2^) using the center method. When difference in ECD-Ce and ECD-Au was plotted against ECD-Ce, a regression formula obtained was: ECD-Au—ECD-Ce = 1028.5–0.397*ECD-Ce, *r* = −0.815, and *R*^2^ = 0.664, *P* < 0.001 (Fig. 3). The difference was 0 when ECD-Ce = 2593 /mm^2^.

**Fig. 4:**
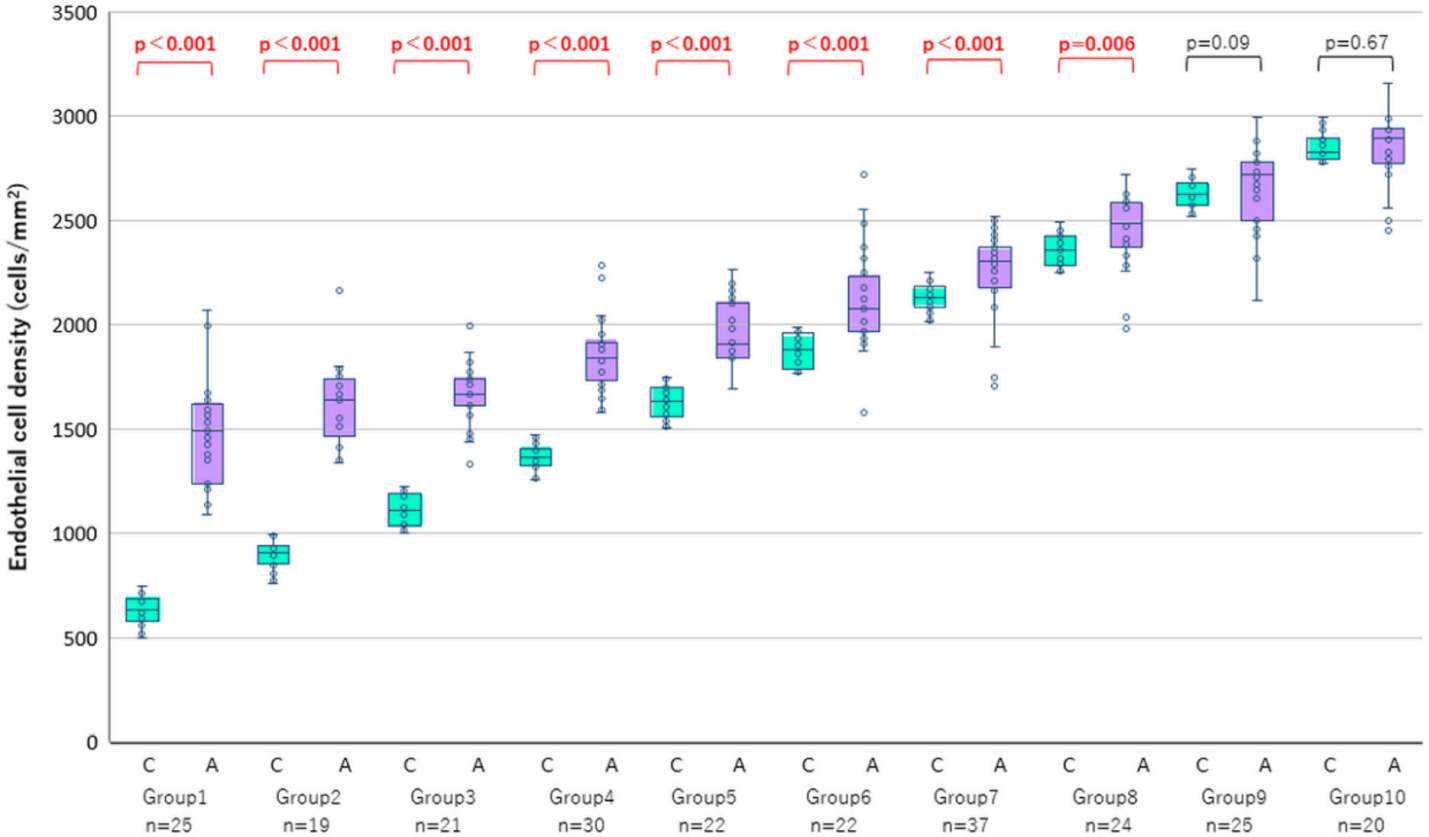
Comparison of the endothelial cell density (ECD) measured by automated and center methods. Each group was classified according to the ECD-Ce. The boxes show the median, as well as 25% and 75% confidence interval (lower and upper quartiles). The whiskers show 95% confidence interval. The ECD values were higher in the automated method than in the center method for Group 1 to 8 (ECD-Ce: 500–2499 cells/mm^2^; *P* < 0.001 for Group 1–7, *P* = 0.006 for Group 8 by the Mann–Whitney U test). The between-method differences were larger in low cell density groups. *n*, number of subjects in each group. *C*, center method; *A*, automated method

Table 3 and Fig. 4 present the ECD data for each group. Compared with ECD-Ce, ECD-Au was significantly higher in Groups 1–8 (ECD-Ce: 500–2499 cells/mm^2^). The *P* value was < 0.001 for Groups 1–7 and *P* = 0.006 for Group 8. The respective between-method differences in the median ECD values were 860, 731, 558, 476, 275, 198, 176, and 127 cells/mm^2^, with the differences being larger in low cell density groups. When the ECD-Ce exceeded 2500 cells/mm^2^, there were no between-method differences in ECD. This suggests that the critical ECD value where the automated method becomes reliable is 2500 cells/mm^2^.

**Table 3:** Endothelial cell density (cells/mm^2^) data in each group classified on the basis of ECD values measured by center method

Group	ECD range (center method)	Median (IQR)			
		Center	Automated	Difference	P*
1 (*n* = 25)	500–749	630 (576–686)	1490 (1235–1616)	860	< 0.001
2 (*n* = 19)	750–999	908 (858–945)	1639 (1463–1738)	731	< 0.001
3 (*n* = 21)	1000–1249	1106 (1038–1188)	1664 (1610–1739)	558	< 0.001
4 (*n* = 30)	1250–1499	1363 (1323–1408)	1839 (1734–1917)	476	< 0.001
5 (*n* = 22)	1500–1749	1630 (1559–1698)	1905 (1844–2104)	275	< 0.001
6 (*n* = 22)	1750–1999	1880 (1788–1960)	2077 (1970–2229)	198	< 0.001
7 (*n* = 37)	2000–2249	2128 (2083–2183)	2304 (2179–2370)	176	< 0.001
8 (*n* = 24)	2250–2499	2358 (2282–2423)	2485 (2369–2586)	127	= 0.006
9 (*n* = 25)	2500–2749	2625 (2571–2681)	2717 (2500–2778)	92	= 0.09
10 (*n* = 20)	2750–2999	2829 (2793–2892)	2895 (2774–2943)	66	= 0.67
Control (*n* = 36)	1961–3322	2667(2344–2877)	2689(2451–2778)	22	= 0.848

^*^P value analysis were made by Mann–Whitney U test

*IQR* interquartile range

**Fig. 5:**
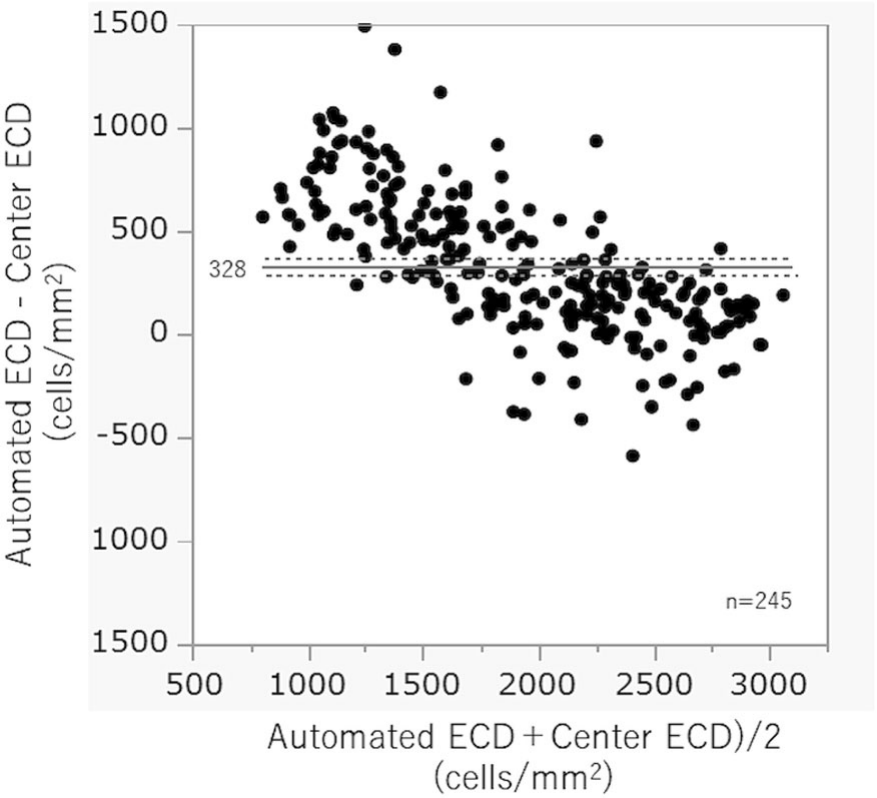
Bland–Altman plots showing between-method comparisons of the endothelial cell density (ECD) of glaucomatous eyes. The solid horizontal line shows the mean difference, while the dashed horizontal lines show the 95% limits of agreement. The mean difference was + 328 cells/mm^2^ (*P* < 0.001). The upper and lower limits of agreement were + 371 cells/mm^2^ and + 286 cells/mm^2^, respectively. There was a negative relationship between the ECD value and the between-method difference, which is indicative of proportional bias

Figure 5 presents the Bland–Altman plots of the between-method comparisons of the ECD value, which showed that the ECD-Au overestimated the ECD by a mean difference of + 328 cells/mm^2^ (*P* < 0.001). Most of the values were not included within the limits of agreement. The differences greatly exceeded the upper limit of agreement for the low ECD groups. An inclination in the scatter of the dots is indicative of a proportional bias.

**Fig. 6:**
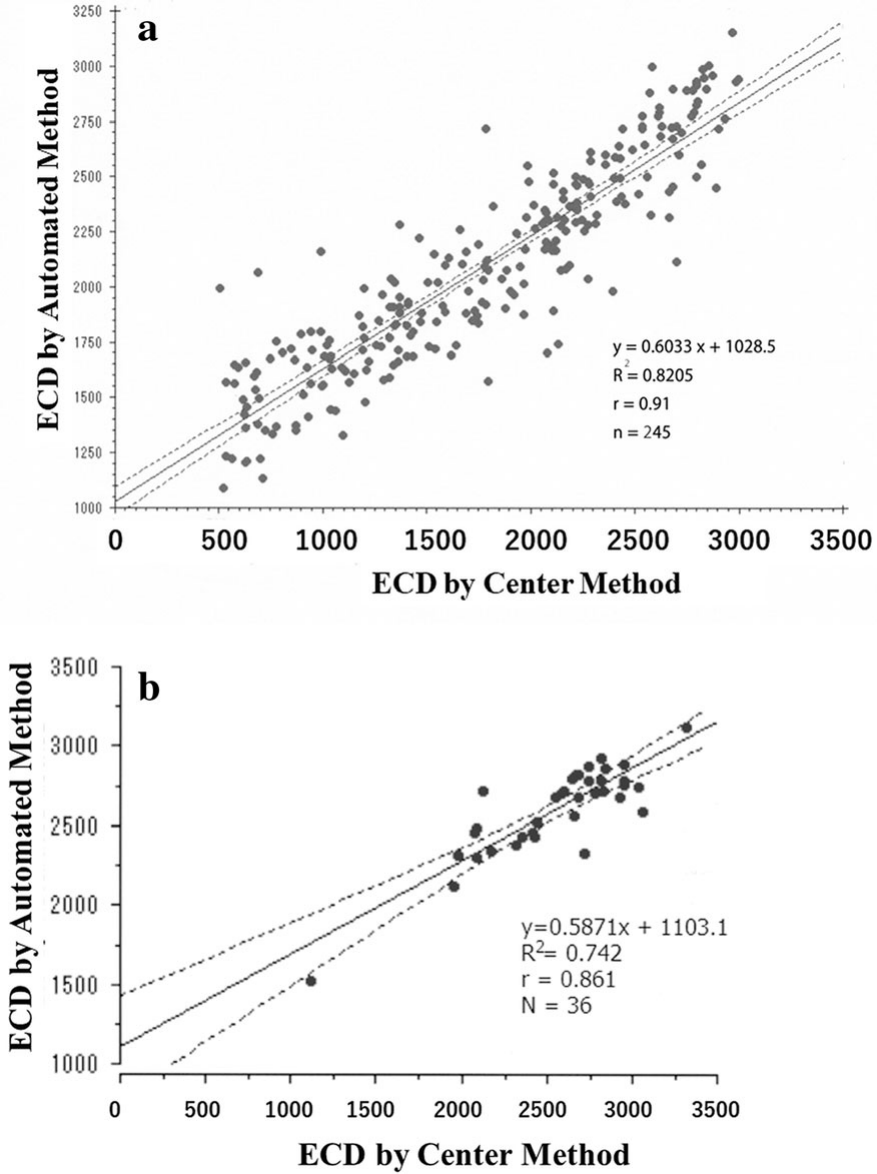
**a** Scatter plot of the endothelial cell density (ECD) by center and automated methods in glaucoma eyes. Solid line indicates regression line, and dotted lines indicate 95% confidence interval. Spearman test showed a close correlation *r* = 0.91, *R*^2^ = 0.8205, and *P* < 0.001, and the linear regression equation was: (ECD-Au, cells/mm^2^) = 0.6033 (ECD-Ce, cells/mm^2^) + 1028.5 cells/mm^2^. **b**. Scatter plot of the endothelial cell density (ECD) by center and automated methods in control eyes. Solid line indicates regression line, and dotted lines indicate 95% confidence interval. Spearman correlation coefficient was *r* = 0.861, *R*^2^ = 0.742, and *P* < 0.001. The linear regression equation of (ECD-Au, cells/mm^2^) = 0.5871 (ECD-Ce, cells/mm^2^) + 1103.1 cells/mm^2^ was quite similar to that in glaucoma eyes (Fig. 6a)

Figure 6a presents a scatter graph showing the correlations between ECD-Au and ECD-Ce in glaucomatous eyes. The correlation coefficient was *r* = 0.91, *R*^2^ = 0.8205, *P* < 0.001, and the linear regression equation was ECD-Au = 0.6033 × ECD-Ce + 1028.5 cells/mm^2^. Although there were significant between-method differences in the ECD values, there was a close correlation in the ECD values of both methods. When the correlation between the ECD-Ce and ECD-Au was studied in the 36 control eyes, the correlation coefficient was high again (*r* = 0.861, *R*^2^ = 0.742, *P* < 0.001). The linear regression equation was ECD-Au = 0.5871 × ECD-Ce + 1103.1 cells/mm^2^. This equation was nearly equal to that in the glaucomatous eyes (Fig. 6b). Thus, close correlation and overestimation of ECD in eyes with low ECD are a common finding in both glaucoma and control subjects.

Table 4 shows the age, IOP, CCT, CV, and HEX values in each group. The Mann–Whitney U test showed significant between-method differences in the CV and HEX values. In all groups, the automated method yielded higher CV values and lower HEX values than those obtained using the center method (*P* < 0.001).

**Table 4:** Age, IOP, and endothelial parameters in each group

Group, ECD (cells/mm^2^) *							
	Age (y) Median (IQR)	IOP (mmHg) Mean (± SD)	CCT (μm) Mean (± SD)		Center Median (IQR)	Automated Median (IQR)	P**
1 (*n* = 25), 500–749				CV	33 (28–38)	60 (57–66)	< 0.001
	74 (66–80)	15.2 ± 4.6	598 ± 64	HEX	54 (47–58)	29 (28–34)	< 0.001
				NUM	24 (21–29)	64 (55–75)	< 0.001
2 (*n* = 19), 750–999				CV	34 (29–37)	54 (51–59)	< 0.001
	66 (49–81)	19.9 ± 12.2	593 ± 82	HEX	52 (44–60)	32 (27–38)	< 0.001
				NUM	35 (27–37)	65 (60–74)	< 0.001
3 (*n* = 21), 1000–1249				CV	33 (29–37)	52 (50–56)	< 0.001
	69 (53–76)	17.2 ± 7.3	575 ± 66	HEX	57 (49–67)	33 (30–39)	< 0.001
				NUM	44 (38–53)	78 (64–79)	< 0.001
4 (*n* = 30), 1250–1499				CV	34 (31–38)	45 (43–52)	< 0.001
	70 (54–76)	16.0 ± 6.2	569 ± 44	HEX	60 (53–62)	35 (32–40)	< 0.001
				NUM	66 (56–69)	85 (79–91)	< 0.001
5 (*n* = 22), 1500–1749				CV	32 (30–38)	48 (44–53)	< 0.001
	69 (41–79)	19.2 ± 7.2	582 ± 66	HEX	58 (51–65)	37 (33–41)	< 0.001
				NUM	59 (49–68)	91 (80–98)	< 0.001
6 (*n* = 22), 1750–1999				CV	34 (31–36)	49 (41–55)	< 0.001
	73 (65–77)	21.5 ± 14	538 ± 103	HEX	62 (56–65)	40 (35–45)	< 0.001
				NUM	63 (43–88)	102 (84–105)	< 0.001
7 (*n* = 37), 2000–2249				CV	36 (32–40)	44 (40–50)	< 0.001
	69 (64–77)	22.4 ± 12.4	559 ± 39	HEX	60 (56–65)	46 (40–50)	< 0.001
				NUM	92 (76–105)	117 (107–122)	< 0.001
8 (*n* = 24), 2250–2499				CV	34 (29–38)	42 (39–48)	< 0.001
	67 (50–75)	16.8 ± 5.7	558 ± 43	HEX	64 (58–69)	43 (39–51)	< 0.001
				NUM	78 (58–107)	124 (118–131)	< 0.001
9 (*n* = 25), 2500–2749				CV	35 (30–36)	41 (385–47)	< 0.001
	70 (61–75)	22.0 ± 9.4	591 ± 44	HEX	60 (55–67)	45 (41–51)	< 0.001
				NUM	94 (56–118)	141 (122–146)	< 0.001
10 (*n* = 20), 2750–2999				CV	36 (34–37)	44 (41–47)	< 0.001
	68 (56–73)	25.1 ± 14.6	581 ± 45	HEX	59 (56–64)	45 (42–47)	< 0.001
				NUM	111 (83–157)	147 (139–155)	< 0.001
Control(n = 36), 1961–3322				CV	35(31–38)	47(41–52)	< 0.001
	74(67–76)	17.8 ± 2.4	567 ± 43	HEX	59(56–63)	43(39–49)	< 0.001
				NUM	91(72–117)	135(120–146)	< 0.001

^*^ECD values were measured by center method. **P value analysis were made by Mann–Whitney U test

*ECD* endothelial cell density, *IQR* interquartile range, *SD* standard deviation, *IOP* intraocular pressure, *CCT* central corneal thickness, *CV* coefficient of variation in cell area, *HEX* percentage of hexagonal cells, *NUM* number of cells analyzed

**Fig. 7:**
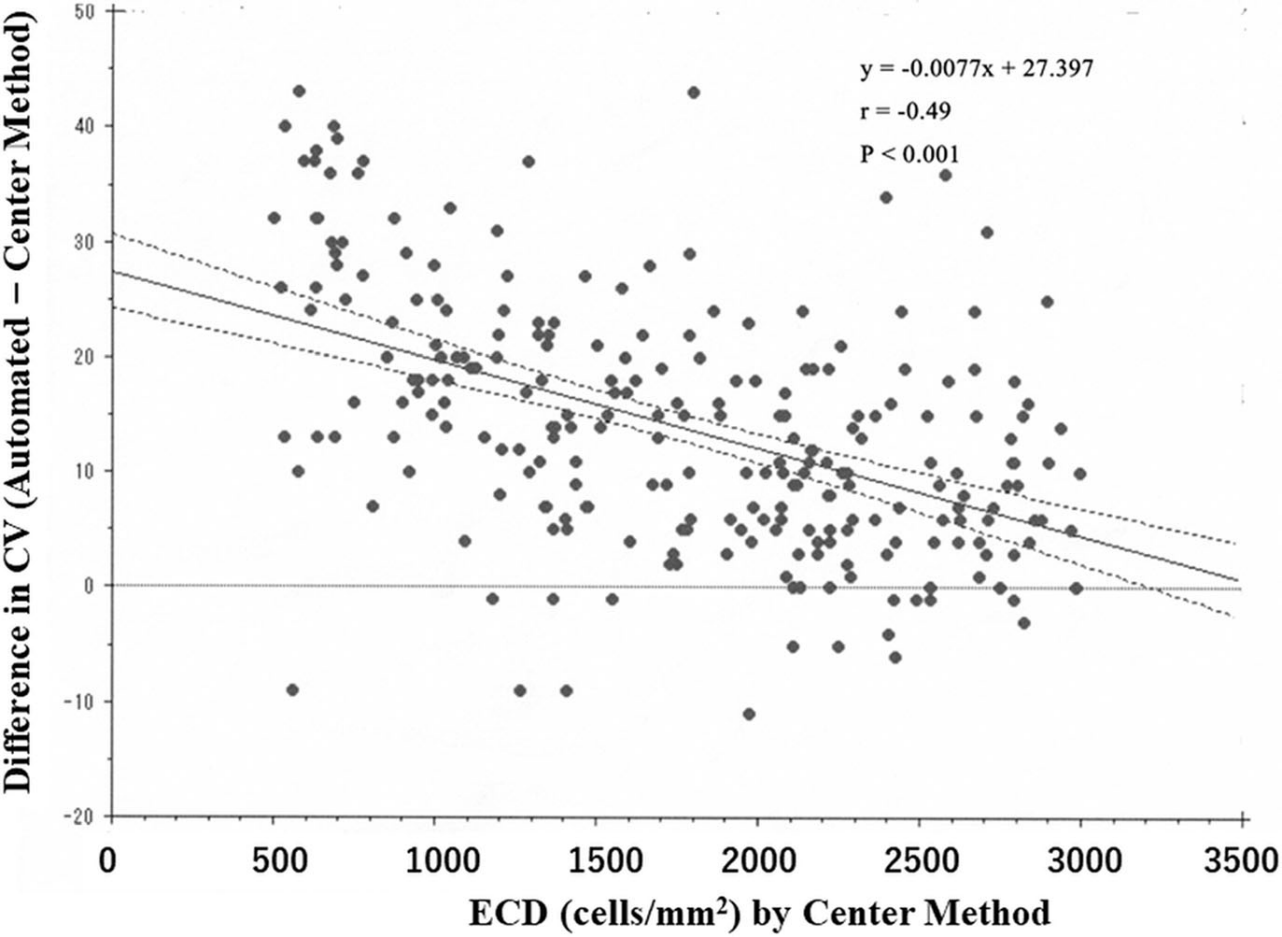
Scatter plot showing between-method differences in the coefficient of variation (CV) against the ECD-Ce with regression line and 95% confidence interval. Compared with the CV value in the center method, the CV was greater in the automated method; moreover, the between-method CV deviation increased with decreased ECD values. Spearman’s correlation analysis showed a negative correlation between the center-automated difference in the CV and ECD-Ce (*r* = −0.49, *P* < 0.001)

**Fig. 8:**
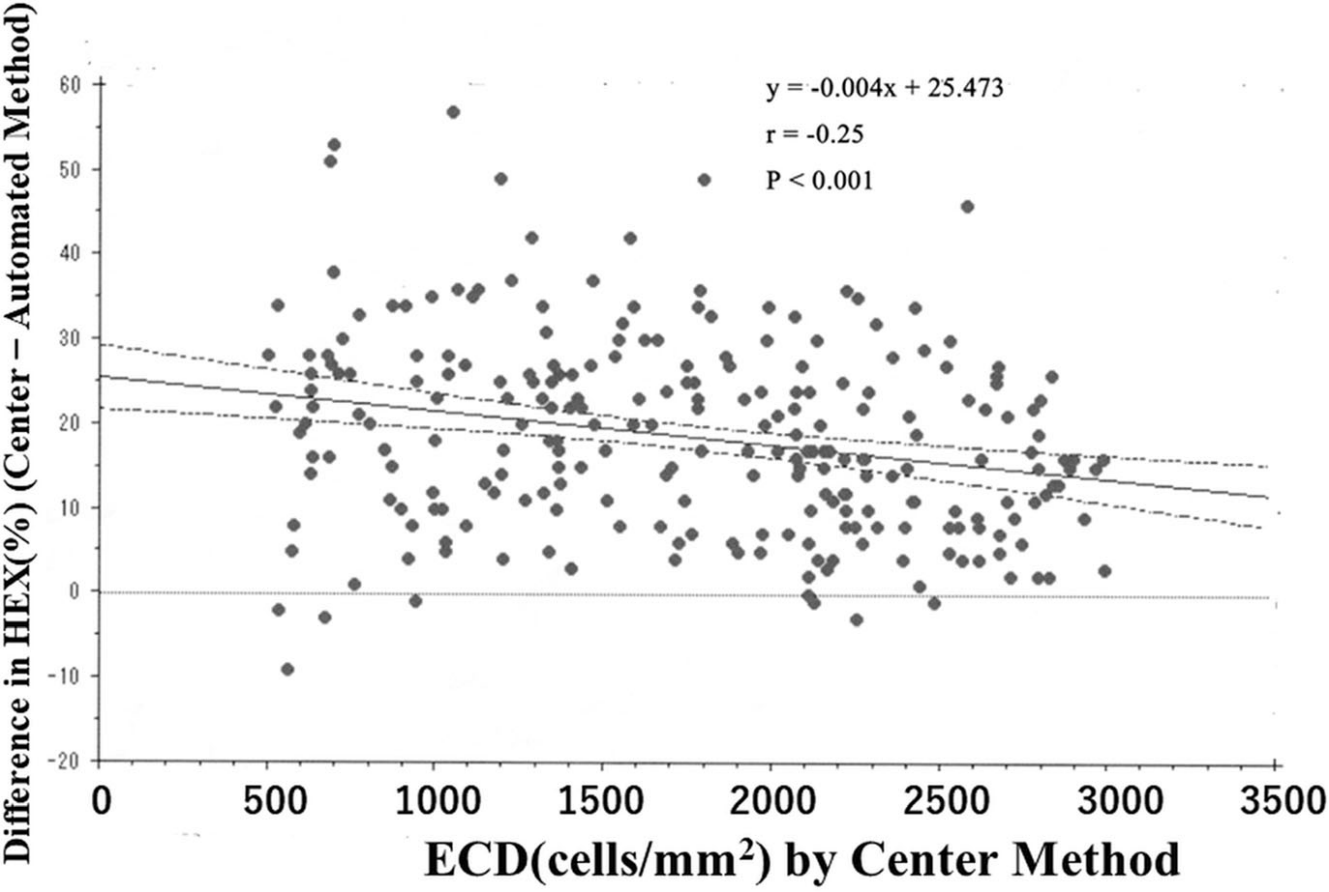
Scatter plot showing between-method differences in the percentage of hexagonal cells (HEX) against the ECD-Ce. Regression line and 95% confidence interval also were shown. The HEX was greater in the center method than in the automated method; moreover, the between-method deviation in the HEX increased with decreased ECD values. Spearman’s correlation analysis showed a negative correlation between center-automated difference in the HEX and ECD-Ce (*r* = −0.25, *P* < 0.001)

Figure 7 shows an increasing tendency of the between-method CV deviation with decreasing ECD-Ce values. Figure 8 demonstrates the increased between-method differences in HEX with decreased ECD-Ce. Between-method differences in CV and HEX were negatively correlated with ECD-Ce (*r* = −0.49, *P* < 0.001 and *r* = −0.25, *P* < 0.001, respectively). This indicates that the differences in CV and HEX were larger at lower ECD values.

**Fig. 9:**
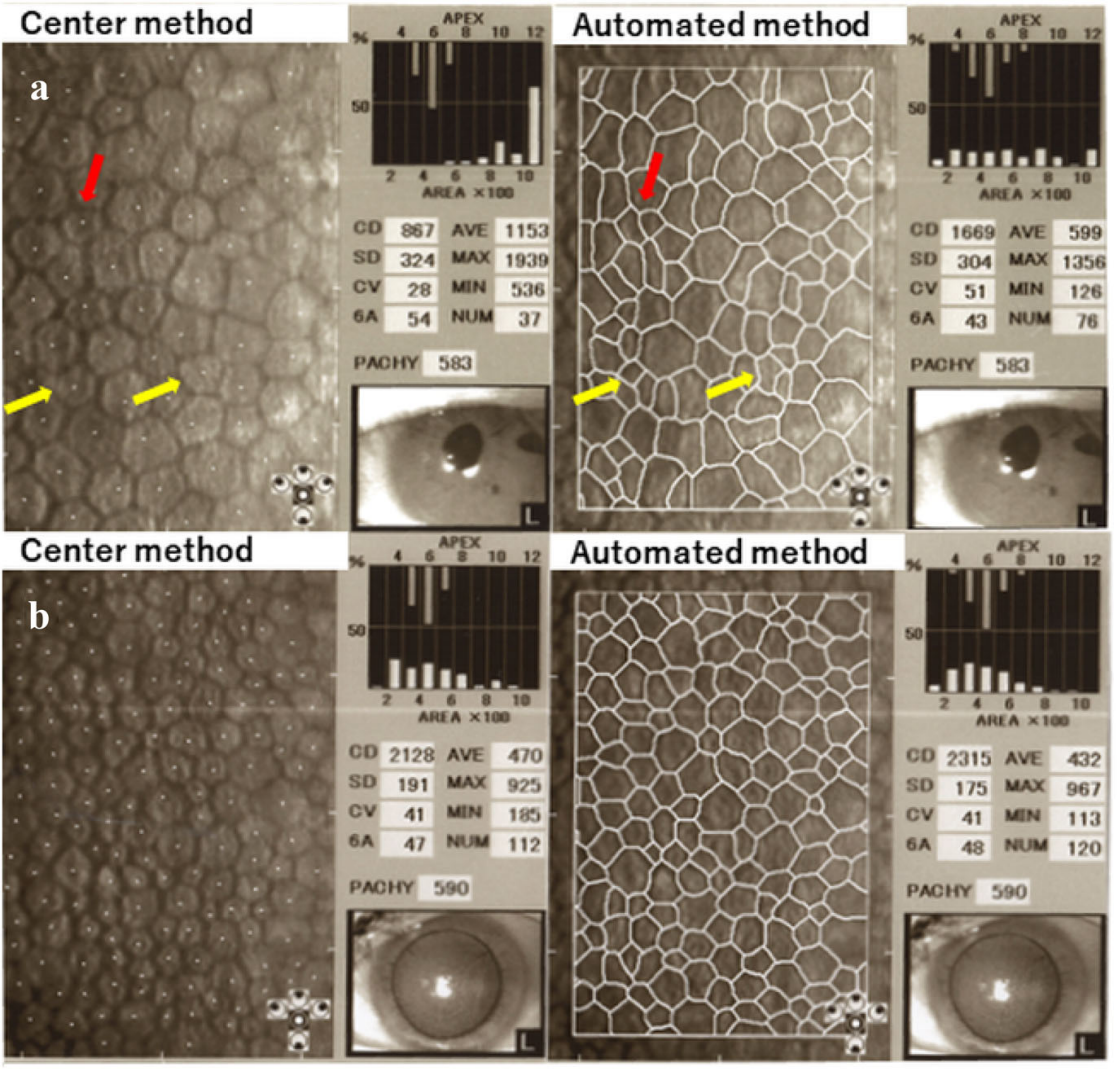
Example of corneal endothelial images that shows between-method differences in the ECD values. **A**: Images obtained from a 70-year-old-man with childhood glaucoma in Group 2. The intraocular pressure (IOP) was 15 mmHg. There were large between-method differences in the endothelial cell density (ECD) and coefficient of variation in cell area (CV) by the center and automated method, which were 867 and 1669 cells/mm^2^ and 28% and 51%, respectively. The automated method misrecognized lines of intracellular structures (yellow arrows) or nucleus (red arrow) as cellular borderlines, which led to the division of large cells to multiple small cells. **B**: Images obtained from a 65-year-old woman with exfoliation glaucoma in Group 7. Her IOP was 31 mmHg. The ECD was slightly higher in the automated method (2315 cells/mm^2^) than in the center method (2128 cells/mm^2^. In Group 7, the between-method deviation in ECD was smaller than that in Group 2; however, there was still a significant difference (*P* < 0.001, Table 2)

Figure 9 shows representative corneal endothelial images where significant between-method differences in the ECD values were observed. Images obtained from a 70-year-old-man in Group 2 revealed ECD values of 867 cells/mm^2^ and 1669 cells/mm^2^ and a CV of 28% and 51% using the center and automated methods, respectively. The automated method misidentified the cell border and erroneously divided one large cell into many small cells (Image A). Image B was obtained from a 65-year-old woman in Group 7 with ECD values of 2128 cells/mm^2^ and 2315 cells/mm^2^ by the center and automated methods, respectively. Even though the between-method deviation in the ECD decreased with increasing ECD-Ce, the between-method difference was still significant in Group 7 (*P* < 0.001).

## Discussion

Few studies have reported differences in the endothelial parameters of glaucomatous eyes between the center and automated methods. Several studies have reported that the fully automated analysis significantly overestimates ECD values in individuals with low ECD [5–7]. Huang et al. compared corneal endothelial parameters between both methods in 106 glaucomatous eyes [5]. However, they did not classify the subjects into smaller ECD-based groups. To the best of our knowledge, there has been no study on the specific ECD level where a significant between-method difference emerges.

The difference between ECD-Ce and ECD-Au is 0 when ECD-Ce = 2593. However, there is a variation in data, and the compatibility between ECD-Ce and ECD-Au is slightly wider and is held if ECD-Ce is 2500 or higher (Figs. 3 and 4). When ECD-Ce was less than 2500 cells/mm^2^, we found significant between-method differences (Table 2, Fig. 3). Therefore, an ECD-Ce of 2500 cells/mm^2^ appears to be the critical ECD level for scientific evaluation using the automated method.

ECD overestimation in damaged corneas may be clinically problematic. Assuming a 0–30% cell loss after intraocular surgery, patients should have an ECD of at least 1000–1200 cells/mm^2^ for safety purposes to reduce the risk of postoperative corneal edema [2, 10]. The true ECD of eyes with pre-surgical ECD-Au of 1500 cells/mm^2^, which is estimated by the regression equation (Fig. 6a), is close to 782 cells/mm^2^. This finding suggests that the eyes with ECD of 1500 by the automated method have a high risk of corneal decompensation and may not tolerate glaucoma surgery.

ECD is an important parameter for assessing endothelial function and health. However, there is a need to assess changes in other parameters. Theoretically, polymegathism determined by CV and pleomorphism (cell shape variations represented by HEX) have been suggested as a more sensitive measure of the endothelium under stress [2]. Corneas with a CV > 40% or the presence of < 50% HEX should be considered abnormal and have an increased risk of postoperative edema [2].

In this study, there were further concerns regarding the reliability of CV values obtained using the automated method since they exceeded 40% in all 10 groups. Moreover, these values were higher than those measured using the center method, especially with low ECD values. Furthermore, HEX values obtained using the automated method were < 50% in all 10 groups, which is considered abnormal. In our study, the between-method deviation in the HEX exceeded 14% in the subgroup eyes with ECD > 2000 cells/mm^2^.

As shown in Image A (Fig. 9), the automated method often misrecognized intracellular structures or endothelial cell nuclei as cellular borders and misidentified large cells as multiple small cells. This caused an overestimation of ECD and CV by the automated method in the low ECD group. This kind of CV overestimation by the automated method tends to occur in individuals with low ECD with damage-induced polymegathism.

Concerning the system of fully automated methods, there is another problem that needs to be addressed. The previous models of the Konan specular microscope had the option of selecting patterns of cell size (S, M, L and XL). However, recent models, including the machine we have used in this study, have no such option and have a default setting of S cell size. As described in previous studies [4, 5], the setting of S cell size is not appropriate in corneas with low ECDs, because this would cause misidentifying the cells as being smaller than they really were and overestimating the cell density. This may be caused by the inherent assumption of the automated method that all endothelial cells in the frame are of uniform cell size. When polymegathism is present, this assumption may lead to incorrect counting. Price et al. reported that even with appropriate use of the sizing feature, the Konan Robo automated software significantly overestimated ECD in the Descemet stripping endothelial keratoplasty eyes [6]. However, in the latest model of the Konan specular microscope, the automated analysis algorithm has been refurbished and may have higher precision in the analysis results. The precision of the refurbished automated analysis will be a subject for future studies. Recently, several studies have reported precise and accurate results using deep learning automatic segmentations of corneal endothelial cell images [10–13]. We expect the development of new software with precise automated analysis in the near future.

There have been various recent programs for corneal endothelial cell analysis in specular microscopy, with each having its advantages and disadvantages. The center method has been approved by the FDA for use in clinical trials [1, 3]. However, the center method excludes the outermost digitized cells of a contiguous group, which leads to a decrease in the number of analyzed cells and an increase in CV. The flex-center method allows the inclusion of the outermost cells and increases the NUM (the number of analyzed cells) and may be recommended for cases with small visible cell numbers [4]. However, the flex-center method requires manual boundary tracing of the contiguous cell in addition to cell-center dotting and may be more time consuming than the traditional center method. Doughty et al. studied the appropriate cell count required to obtain accurate ECD, and they recommended a cell count > 75; however, they only assessed the normal cornea (average ECD = 3519 cells/mm^2^) [14]. Individuals with low ECD may have only a few countable cells. Huang and others reported that in corneas with guttae, the center method yields an accurate ECD value when there are ≥ 30 contiguous countable cells in an image. If the number of countable cells is less than 30, the flex-center method may be more suitable [15], and a minimum of 30 cells may be appropriate to obtain an accurate ECD value using the center method.

In conclusion, the fully automated method for glaucomatous eyes significantly overestimates the cell count in eyes with low ECD. An ECD of 2500 cells/mm^2^ is a critical cell count where overestimation occurs in the automated method. The ECD of 1500 using the automated method does not warrant that the eye can tolerate glaucoma surgery.

## Limitation of this study

Although we carefully analyzed images and studied intra-individual deviations, the center method could vary across examiners. We did not determine inter-individual variations, and it is a subject for future studies. This study was conducted using only one type of non-contact specular microscope; thus, the results may not apply to other machines.

## Data Availability

Available on request.
